# The relationship between atopic dermatitis and atopic itch in children and the psychosocial functioning of their mothers: A cross-sectional study

**DOI:** 10.3389/fmed.2023.1066495

**Published:** 2023-02-16

**Authors:** Aleksandra K. Kobusiewicz, Bartlomiej Tarkowski, Andrzej Kaszuba, Aleksandra Lesiak, Joanna Narbutt, Anna Zalewska-Janowska

**Affiliations:** ^1^Psychodermatology Department, Pulmonology, Rheumatology and Clinical Immunology Chair, Medical University of Lodz, Lodz, Poland; ^2^Department of Dermatology, Pediatric Dermatology and Dermatological Oncology, Medical University of Lodz, Lodz, Poland

**Keywords:** atopic dermatitis, itch, mothers, quality of life, sleep, stress

## Abstract

Atopic dermatitis is a chronic inflammatory skin disease significantly affecting patients’ and their parents’ lives. Mothers are mostly responsible for the long-term treatment and their wellbeing is essential. The major objective of this cross-sectional study was to investigate the relationship between atopic dermatitis in children, especially concomitant itch, and the quality of life, stress, sleep quality, anxiety, and depression of their mothers. The study included 88 mothers of children with atopic dermatitis and 52 mothers of children without atopic dermatitis. All mothers completed sociodemographic questionnaire, the Perceived Stress Scale, the Athens Insomnia Scale and the Hospital Anxiety and Depression Scale. Additionally, mothers of children with atopic dermatitis filled in the Family Dermatology Life Quality Index. The severity of atopic dermatitis and pruritus intensity were evaluated by the Scoring Atopic Dermatitis Index and the Numerical Rating Scale, respectively. The severity of atopic dermatitis and itch significantly correlated with the quality of life, insomnia, and perceived stress of the mothers. Mothers whose children had had atopic dermatitis for more than 6 months had significantly higher scores of anxiety and depression. The results highlight the importance of screening mothers for functional impairment to provide adequate support. More attention should be directed to the standardization of stepped care interventions addressing factors resulting in the impaired functioning of mothers.

## 1. Introduction

Atopic dermatitis (AD) is the most common chronic, pruritic, inflammatory skin disease in pediatric patients and often presents as ill-defined, erythematous weeping or crusted papules and plaques. It affects up to 20% of preschool children, with increasing prevalence in developed countries (1). AD in children has been extensively investigated, especially regarding its influence on the functioning of the patients and their parents, and found to be a debilitating disease due to its highly negative physical and mental consequences (2).

Out of all of the symptoms of AD, itch is reported as the most burdensome, affecting almost 91% of patients on a daily basis (3). Sleep disturbance is an important consequence of itch in children. It has been associated with daytime fatigue, irritation, loss of concentration, headaches and increased rates of attention deficit hyperactivity disorder (4). In adults, the “itch that rashes” is believed to drive much of the impact on the quality of life (QoL) and to increase mental distress, leading to a higher risk of suicidal ideation, anxiety, and depression (5). Sleep disturbance in the course of AD begins early in infancy and often leads parents to co-sleep with their infants to prevent them from constantly scratching themselves (6). Managing night-time pruritus results in regular sleep loss for parents and leads to tiredness, increased marital tension, impaired occupational functioning, and a higher rate of anxiety and depression (7). Reducing itch has been found to be the most important treatment goal (8).

Moderate to severe forms of AD in children negatively influence the emotional life of their caregivers. Parents of children with AD report feeling helpless and distressed about caring for them (9). They tend to become overprotective and develop more empathy toward their children, often feeling guilty and blaming themselves for the child’s disease and related suffering (10). Furthermore, parents of children with AD report inadequate social support or even receiving criticism for their parenting from relatives and society (10, 11).

Although the AD of a child influences the psychosocial functioning of both parents, it has a greater impact on the mother’s QoL than on the father’s (12). Current knowledge supports interdisciplinary approach to improve the wellbeing of patients and their caregivers based on the “greater patient” concept (13). Applied from the onset of AD, it could prevent patients and their caregivers from experiencing the considerable burden of AD. It is known that genetic, personal, and environmental variables (e.g., locus of control, coping stress strategies, social support, and various psychological traits) may predict, protect against, and either maintain or counteract anxiety, depression, and perceived level of stress amongst parents of children with AD. Therefore, it is important to investigate which factors impact wellbeing of primary caregivers and how they do so, to better tailor programs supportive to their needs.

The aim of this cross-sectional study was to compare perceived stress levels, sleep patterns, depression, and anxiety in mothers of children with and without AD. Moreover, the major objective of this study was to investigate the relationship between AD, with particular emphasis on pruritus, and the psychosocial functioning of the mothers of affected children.

## 2. Materials and methods

The study included mothers of children with AD and mothers of children without AD for comparison. Children with AD were hospitalized due to AD exacerbation in the Department of Dermatology, Pediatric Dermatology, and Oncology of the Medical University of Lodz, Poland. Children without AD visited the same Department of Dermatology on an outpatient basis or attended a preschool located in Lodz.

Inclusion criteria were the following: Age of the child from 3 months to 18 years, age of the mother of at least 18 years, and agreement to participate in the study. AD was diagnosed according to Hanifin and Rajka. Exclusion criteria included additional chronic diseases in children and psychiatric or other chronic disorders in mothers.

The study was approved by the Bioethics Committee of the Medical University of Lodz (RNN/296/17/KE) and was performed according to the principles of the Helsinki Declaration. All participants provided written informed consent.

The severity of AD in the children was evaluated with the Scoring Atopic Dermatitis (SCORAD) index by an experienced dermatologist (AKK) (14). Mothers of children with AD were asked to rate their child’s itch intensity from the past 3 days on a numerical rating scale (NRS) and to complete a questionnaire with sociodemographic data and questions about the onset and duration of AD, and the Polish-language versions of the Family Dermatology Life Quality Index (FDLQI), the Perceived Stress Scale (PSS 10), Athens Insomnia Scale (AIS), and Hospital Anxiety and Depression Scale (HADS). Mothers of children without AD were asked to fill in the sociodemographic questionnaire, PSS 10, AIS, and HADS.

The modified NRS used in the study is a 10-cm long horizontal line with numbers from 0 to 10 on which participants indicate the intensity of pruritus, with 0 being no pruritus and 10 being the worst itch. Itch NRS scoring was categorized as mild (>0–3 points), moderate (≥3–7 points), and severe or very severe itch (≥7–10 points) (15–17).

The FDLQI measures the impact of the children’s skin disease on the caregivers’ QoL in the past month (18). The questionnaire consists of 10 questions concerning the influence of the patient’s skin disease on different aspects of family life. Each question can be answered by choosing 1 out of 4 answers scored 0–3. Higher scores indicate poorer QoL (19).

The PSS 10 measures perceived level of stress in the participant’s life during the past month (20). It is a 10-item questionnaire that participants answer on a 5-point scale ranging from 0 (never) to 4 (very often). The higher the score, the greater the perceived stress. The Polish version scale has a Cronbach’s α of 0.86 (20).

The AIS is a self-rated psychometric questionnaire measuring sleep disturbances over the past month, based on the criteria of the International Classification of Diseases—10th edition (ICD-10). Each of the eight questions can be scored on a 0–3 scale, in which three designates negative outcomes. Scores ≥6 points reflect a diagnosis of insomnia (16, 21).

The HADS is used to identify anxiety and depression symptoms during last week (22). It consists of 14 questions divided in two subscales for anxiety (HADS-A) and depression (HADS-D). Each question can be answered on a scale ranging from 0 (never) to 3 (very often). The maximum score for each subscale is 21. Scores indicating probable anxiety/depression were both defined as ≥8.

### 2.1. Statistical analysis

The results are presented as means ± standard deviations (SDs). The data were analyzed with descriptive statistics, using non-parametric tests because the data did not meet the assumptions about normal distribution and equality of variances. Correlations were determined using Spearman’s rho. Groups that differed in disease duration where analyzed using the Mann–Whitney *U*-test. The level of significance was set at α = 0.05. Statistical analyses were performed using Jasp ver.0.12.1/774.

The sample size of the study cohort was determined by sample size calculation using the principle of the anticipated response distribution of 50%, with 95% confidence interval (CI), and 10% precision.

## 3. Results

A total of 120 mothers of children with AD and 70 mothers of children without AD were found to be eligible for the study. However, 10 mothers of children with AD and 12 mothers of children without AD refused consent due to lack of time. In addition, 22 and 6 mothers, respectively, did not return the completed tests. Thus, the study group consisted of 88 mothers of children with AD (response rate 73.33%), whereas the control group comprised 52 mothers of children without AD (response rate 74.29%). The characteristics of the study and control groups, including sociodemographic data, AD disease parameters, and overall psychosocial health status are summarized in Table 1.

**TABLE 1:** Characteristics of the study group.

	Mothers of children with AD, *n* = 88	Mothers of children without AD, *n* = 52	*P*-value	Children with AD, *n* = 88	Children without AD, *n* = 52	*P*-value
Age of mother, years, mean ± SD	35.05 ± 6.56	34.81 ± 3.88	0.795	N/A	N/A	
Range	19–52	27–42				
Age of children, months, mean ± SD	N/A	N/A		60.16 ± 56.60	60.25 ± 37.71	0.227
Range				1–216	5–132	
Sex of child	N/A	N/A				0.006
Male, *n* (%)				59 (67.05)	29 (55.77)	
Female, *n* (%)				29 (32.95)	23 (44.23)	
Education			0.051	N/A	N/A	
<9 years of education, *n* (%)	1 (1.14)	2 (3.85)				
High school, *n* (%)	27 (30.68)	7 (13.46)				
Vocational school, *n* (%)	10 (11.36)	3 (5.77)				
University degree, *n* (%)	50 (56.82)	40 (76.92)				
Employment				N/A	N/A	
Employed, *n* (%)	66 (75)	44 (84.62)	0.355			
Unemployed, *n* (%)	22 (25)	8 (15.38)				
Marital status				N/A	N/A	
Single, *n* (%)	2 (2.27)	1 (1.92)	0.809			
Married, *n* (%)	61 (69.32)	38 (73.08)				
Partnership, *n* (%)	16 (18.18)	8 (15.38)				
Divorced, *n* (%)	7 (7.96)	3 (5.77)				
Widowed, *n* (%)	2 (2.27)	0 (0.00)				
Unknown, *n (%)*	0 (0.00)	2 (3.85)				
Average number of children, mean ± SD	1.55 ± 0.71	1.58 ± 0.70	0.706	N/A	N/A	
Range	1–4	1–3				
AD in the family	N/A	N/A				
Mothers, *n* (%)				3 (3.40)	3 (5.77)	
Fathers, *n* (%)				3 (3.40)	1 (1.92)	
Siblings, *n* (%)				7 (7.95)	0 (0.00)	
No, *n* (%)				78 (88.64)	48 (92.31)	
Duration of AD, months, mean ± SD	N/A	N/A		43.68 ± 51.19	N/A	
Range				1–216		
<6 months, *n* (%)				27 (30.68)		
≥6 months, *n* (%)				61 (69.32)		
SCORAD (points), mean ± SD	N/A	N/A		46.64 ± 15.17	N/A	
Range				13–84		
Mild, <25, *n* (%)				11 (12.50)		
Moderate, 25–50, *n* (%)				46 (52.27)		
Severe, >50, *n* (%)				31 (35.23)		
Itch NRS (points), mean ± SD	N/A	N/A		6.14 ± 3.03	N/A	
Range				0–10		
Mild, <3, *n* (%)				11 (12.50)		
Moderate, 3–6, *n* (%)				29 (32.96)		
Severe, 7–10, *n* (%)				48 (54.54)		
FDLQI, (points), mean ± SD	16.45 ± 6.56	N/A		N/A	N/A	
Range	0–30					
PSS-10, (points), mean ± SD	21.66 ± 6.81	16.90 ± 5.83	<0.001	N/A	N/A	
Range	1–35	2–29				
AIS, (points), mean ± SD	9.20 ± 5.46	6.00 ± 3.92	<0.001	N/A	N/A	
Range	0–20	1–16				
Insomnia, ≥6, *n* (%)	59 (67.05)	23 (44.23)				
HADS A, (points), mean ± SD	9.12 ± 4.63	4.63 ± 3.38	<0.001	N/A	N/A	
Range	0–18	0–16				
Anxiety, >8, *n* (%)	51 (57.96)	6 (11.54)				
HADS D, (points), mean ± SD	7.34 ± 4.13	3.40 ± 3.10	<0.001	N/A	N/A	
Range	0–15	0–14				
Depression, >8, *n* (%)	44 (50.00)	4 (7.69)				

AD, atopic dermatitis; N/A, not applicable; SD, standard deviation; SCORAD, scoring of AD; NRS, numerical rating scale; FDLQI, Family Dermatology Life Quality Index; PSS-10, Perceived Stress Scale; AIS, Athens Insomnia Scale; HADS A, Hospital Anxiety and Depression Scale, Subscale Anxiety; HADS D, Hospital Anxiety and Depression Scale, Subscale Depression.

The mean age of mothers of children with and without AD was 35.05 ± 6.56 years (range 19–52 years) and 34.81 ± 3.88 (range 27–42 years), respectively. In the group of children with AD, the mean age was 60.43 ± 56.60 months (range 1–216 months) and about 67% were boys. Children without AD were age-matched, with the mean age of 60.25 ± 37.71 months (range 5–132 months).

Three mothers in each group reported having had AD in the past. The mean duration of AD in children was 43.68 months, with large differences in the duration of the disease. According to the SCORAD index, most of the children had moderate AD (52%), followed by severe (35%). Itch was permanently present in every child with AD, with most of the children (54%) experiencing severe itch according to the NRS assessment.

The children’s AD had a negative impact on various aspects of their mothers’ wellbeing and QoL. Mothers of children with AD reported higher levels of perceived stress compared to the control group (*p* < 0.001). Sixty-seven percent of mothers in the study group and 44% in the control group had co-existing insomnia. Also, 58 and 50% of mothers of children with AD reported symptoms of anxiety and depression, respectively (HADS A and HADS *D* ≥ 8), whereas among controls, the proportions were 11.5% and almost 8%, respectively. Between-group differences in HADS scores are statistically significant (*p* < 0.001).

The correlations between the severity of the children’s AD, the duration of the disease, the intensity of itch and the psychosocial functioning of their mothers are presented in Table 2. The severity of AD in children (SCORAD) correlated significantly with the mothers’ FDLQI (ρ_*s*_ = 0.38, *p* < 0.001), PSS-10 (ρ_*s*_ = 0.26, *p* = 0.02), and AIS (ρ_*s*_ = 0.27, *p* = 0.017) scores but not with their HADS A and HADS D scores. Similarly, statistically significant correlations were found between the severity of itch (NRS) and the FDLQI (ρ_*s*_ = 0.43, *p* < 0.001), AIS (ρ_*s*_ = 0.33, *p* = 0.002), and PSS 10 (ρ_*s*_ = 0.32, *p* = 0.003) scores but not with symptoms of anxiety (HADS A) and depression (HADS D).

**TABLE 2:** Correlation between the parameters of the children’s atopic dermatitis and the psychosocial functioning of their mothers.

	SCORAD ρ _*s*_ (*p*-value)	Itch NRS ρ _*s*_ (*p*-value)	Disease duration ρ _*s*_ (*p*-value)
FDLQI	0.38 (<0.001*)	0.43 (<0.001*)	0.33 (0.002*)
PSS 10	0.26 (0.021*)	0.32 (0.003*)	0.11 (0.293)
AIS	0.38 (0.017*)	0.33 (0.002*)	0.10 (0.377)
HADS A	0.11 (0.32)	0.17 (0.131)	0.20 (0.072)
HADS D	0.13 (0.24)	−0.03 (0.796)	0.17 (0.130)

ρ_s_: Spearman’s rho; *p < 0.05; SCORAD, scoring of atopic dermatitis; NRS, numerical rating scale; FDLQI, Family Dermatology Life Quality Index; PSS-10, Perceived Stress Scale; AIS, Athens Insomnia Scale; HADS A, Hospital Anxiety and Depression Scale, Subscale Anxiety; HADS D, Hospital Anxiety and Depression Scale, Subscale Depression.

Although the duration of the disease did not correlate with the HADS scores, we found that mothers of children with AD presented significantly higher HADS scores when the duration of the disease was longer than 6 months. The scores are presented in Table 3.

**TABLE 3:** Results from the Mann–Whitney *U*-test comparing the HADS scores of mothers whose children had had atopic dermatitis for less than 6 months vs. longer than 6 months.

	Group	*N*	Mean	SD	*U*	*P*-value
HADS A	<6 months	30	7.80	4.23		
	>6 months	56	9.82	4.71	622.00	0.024*
HADS D	<6 months	29	6.17	3.78		
	>6 months	56	7.95	4.21	604.50	0.027*

HADS A, Hospital Anxiety and Depression Scale, Subscale Anxiety; HADS D, Hospital Anxiety and Depression Scale, Subscale Depression, N, number of mothers, SD, standard deviation, U, Mann–Whitney *U*-test value; *p < 0.05.

## 4. Discussion

Our results indicate that the severity of AD and pruritus in children affects various aspects of the psychosocial functioning of their mothers. AD is a burdensome disease for the families of the affected children, and caregivers, in particular, report higher levels of stress than those of children not affected by this disease (23, 24). Mothers are most often the primary caregivers of children with AD (25), and the severity of the illness has a greater impact on the QoL of mothers than of fathers (12, 25). The QoL impairment of the mothers in our study was similar to that reported by others (12, 25), as was their level of perceived stress (25). Our results suggest that the mothers of children with AD have a lower QoL than the caregivers of children with other pediatric dermatoses, including epidermolysis bullosa (26), psoriasis (27), vitiligo (28), and alopecia areata (29).

The mothers’ QoL impairment and level of perceived stress had a significant positive correlation with the severity of AD in their children, which is in agreement with data reported by other authors (25, 30–32). In our study, the QoL was the only psychosocial parameter of the mothers that was correlated with disease duration, suggesting a cumulative negative effect. Interestingly, some have reported that the higher stress in the mother is related to coping mechanisms and family structure rather than to the severity of the disease (24, 33).

In our study insomnia affected a significant percentage of mothers in both the study and the control groups. Several studies documented that sleep in women may be negatively affected by biological, personal and environmental variables (e.g., hormonal changes in female reproductive cycle, life stressors, use of stimulant medications) (34). In women who are mothers insomnia may be also associated with post-partum period, breastfeeding, child sleep patterns and disturbances, and maternal distress of caring for a child (35). Additionally having a child with disease is another important factor of sleep problems (35). We observed a positive correlation between the severity of AD and sleep disturbances in the mothers, similar to what has been reported by others (6, 36). The severity of AD is associated with poor quality of sleep in both the parents and the affected children (6). One reason for this is that the more severe AD, the more severe itch is reported. Itch is the most prominent symptom of AD and has a significant detrimental effect on the patient’s QoL. It generally worsens at night and is responsible for a diminished quality of sleep in patients. In our study, itch was universally present, and most of the children experienced severe itch. Scratching or rubbing the skin indicates the child’s suffering. This may evoke helplessness, distress and irritation in the mothers, who try to prevent the child from scratching eczema (24).

Indeed, we observed that the more severe the itch, the greater the QoL impairment, severity of insomnia, and perceived stress of the mothers. To the best of our knowledge, this is the first study that comprehensively presents the relationship between atopic itch in children and functional impairment of their mothers. Loss of sleep is particularly troublesome in the mothers, since they are also in charge of many other normal parenting activities besides taking care of their child’s AD treatment. To cope with the increased level of itch at night, parents of children with AD often develop shift-sleeping or co-sleeping strategies (2, 6). However, both of these strategies are doubtful. Shift-sleeping is associated with numerous health risks, including diabetes, cardiovascular disease (2), and motor and executive function deterioration. Co-sleeping, on the other hand, leads to unhappiness and increased level of stress in the parents (6).

Others, however, have found that the loss of sleep in the mothers of children with AD is not associated with the child’s sleep disturbances (34). Research in other pediatric chronic illnesses suggest that the sleep disturbances in the caregivers may be related to their stress and anxiety about their child’s illness (36). Moore et al. found that parents of children with AD lost more sleep than parents of children with asthma, and the severity of sleep disturbance was directly correlated with anxiety and depression in the mothers (7). In our study, half of the mothers had symptoms of anxiety and depression, and mothers with children that had had AD for longer than 6 months reported more severe symptoms, which might arise from feelings of guilt or pressure put upon the mothers (32).

Many studies have shown that structured educational programs reduce the severity of AD, parental stress and anxiety, as well as improve the quality of family life and parental disease management (37–39). Although educational programs are recommended in recent guidelines, no consensus has been reached on the form and content (40). Educational strategies and psychological support programs should be developed and used along with conventional therapy (41). Stepped care model that monitors and delivers interventions depending on the level of parental distress or needs could also be beneficial for both parents and patients (42). In some countries, e.g., Germany and the United Kingdom, “atopic schools” with multi-disciplinary teams (including clinicians and psychologists) that offer patient education are becoming increasingly popular (41). In Poland, such centers are still lacking, which might have influenced the mothers’ psychological status.

Our study has a few limitations. It was conducted at a single center in Poland, which limits the generalization of the results to other populations, including those receiving better education about AD. Mothers were recruited from a hospital-based dermatology ward where more severe cases of eczema are more prevalent, and this may also limit the generalization of our findings. Furthermore, children without AD were not sex-matched to those with AD. Additionally, we measured the intensity of itch with the NRS scale, which may not accurately detect the children’s pruritus because their mothers filled in the questionnaire on their behalf. Although it is a valid and widely used instrument to asses pruritus in children, results are subjective and dependent on personal interpretation. However, no single method has been recognized as a gold standard to objectively assess itch intensity in the pediatric population in clinical trials (16).

To summarize, QoL impairment, sleep disturbances, and the perceived level of stress in the mothers of children with AD are correlated with the severity of AD and atopic itch. Furthermore, a prolonged illness can aggravate symptoms of anxiety and depression in the mothers. Mothers are often responsible for the successful treatment of AD in their children by ensuring adherence to medical recommendations and supervising children unable to independently implement a multi-element therapeutic process. The management of children with AD should include effective reduction of pruritus in children, screening for functional impairment of their mothers and the provision of psychological support for them to ensure long-term treatment adherence and prevent further consequences of the disease. More attention should be directed to the standardization of stepped care interventions addressing factors resulting in the impaired functioning of mothers. These interventions should consist of education, training in stress-coping strategies, pharmacological and psychological itch management techniques, supportive groups, and regular mental health support to prevent anxiety and depression.

## Data availability statement

The raw data supporting the conclusions of this article will be made available by the authors, without undue reservation.

## Ethics statement

The studies involving human participants were reviewed and approved by the Bioethics Committee of the Medical University of Łódź. Written informed consent to participate in this study was provided by the participants or their legal guardian/next of kin.

## Author contributions

AKK and AZ-J: conceptualization, methodology, and funding acquisition. AKK: validation, investigation, writing—original draft preparation, visualization, and project administration. BT and AKK: formal analysis. AKK, AK, JN, and AL: resources. AKK and BT: data curation. BT, AZ-J, AL, JN, and AK: writing—review and editing. AZ-J: supervision. All authors have read and approved the published version of the manuscript, guarantee the integrity, and accuracy of this study.
